# A Systematic Video Analysis of Anterior Cruciate Ligament Injuries in Professional Female Basketball Players

**DOI:** 10.1177/03635465251330007

**Published:** 2025-04-06

**Authors:** Kristian Heder Ternell, Filippo Tosarelli, Matthew Buckthorpe, Kristian Samuelsson, Eric Hamrin Senorski, Francesco Della Villa

**Affiliations:** * Department of Orthopaedics, Institute of Clinical Sciences, Sahlgrenska Academy, University of Gothenburg, Gothenburg, Sweden; † Sahlgrenska Sports Medicine Center, Gothenburg, Sweden; ‡ Education and Research Department, Isokinetic Medical Group, Bologna, Italy; § Faculty of Sport, Technology and Health Sciences, St Mary’s University, Twickenham, London, UK; ‖ Department of Orthopaedics, Sahlgrenska University Hospital, Sahlgrenska Academy, University of Gothenburg, Mölndal, Sweden; ¶ Department of Health and Rehabilitation, Institute of Neuroscience and Physiology, Sahlgrenska Academy, University of Gothenburg, Gothenburg, Sweden; Investigation performed at the Department of Orthopaedics, Institute of Clinical Sciences, Sahlgrenska Academy, University of Gothenburg, Gothenburg, Sweden

**Keywords:** anterior cruciate ligament, ACL, video analysis, female, basketball, biomechanics

## Abstract

**Background:**

Anterior cruciate ligament (ACL) injuries are common in female basketball players, who have a 2 to 3 times higher risk for an ACL injury than their male counterparts. Improving our understanding of the situations and biomechanics that result in an ACL injury in basketball players may support the design of more effective programs to mitigate the injury risk.

**Purpose:**

To describe the injury mechanisms, situational patterns, and biomechanics of ACL injuries during matches in professional female basketball players.

**Study Design:**

Case series; Level of evidence, 4.

**Methods:**

A total of 105 ACL injuries in professional female European basketball leagues from the 2018 to 2023 seasons were identified. There were 41 (39%) injury videos analyzed for injury mechanisms and situational patterns, while biomechanical analysis was possible in 33 cases. There were 3 independent reviewers who rated each video. Data according to player position, phase of the match, and location on the court were also evaluated (n = 41). Analysis regarding neurocognitive perturbation was assessed in 41 cases (100%).

**Results:**

Most injuries (n = 28 [68%]) occurred during offensive possession. Indirect contact injuries were more prevalent (n = 23 [56%]) than noncontact injuries (n = 18 [44%]). Overall, 3 main situational patterns constituted 95% of injuries: offensive cut (n = 20 [49%]), defensive cut (n = 12 [29%]), and landing from a jump (n = 7 [17%]). Injuries involving a knee-dominant pattern with valgus were identified in 64% of cases. Injuries were evenly distributed between the first (53%) and second (47%) halves of a game. Half (50%) occurred within the first 10 minutes of effective playing time, and the most frequent months of injury were October and November (44%; early season). Nearly half (46%) of the injuries occurred in small forwards, with 59% of injuries occurring in zone 5. Neurocognitive errors were seen in 56% of injuries, while 92% of defensive injuries showed signs of neurocognitive errors.

**Conclusion:**

ACL injuries in professional female basketball players were caused by indirect contact or noncontact, involving offensive and defensive cuts as well as landing from a jump. Biomechanical analysis confirmed a multiplanar mechanism with a knee-dominant pattern and valgus. Most injuries occurred early in the season and within the first 20 effective minutes played (89%), making accumulated fatigue an unlikely risk factor for ACL injuries in basketball players.

Anterior cruciate ligament (ACL) injuries are challenging for female basketball players largely because of the long return-to-play time, which ranges from 9 to 13 months.^1,22^ Female players are 3.5 times more likely to suffer an ACL tear than their male counterparts,^26^ and 20% of returning athletes experience a second tear.^38^ Athletes who have previously undergone ACL reconstruction are 6 times more likely to sustain a second ACL injury.^6^ ACL injuries are common, accounting for 37% of total knee injuries,^1^ and ACL reconstruction is the most frequent surgical procedure among Women’s National Basketball Association (WNBA) players.^19^ Additionally, there is an increased rate of secondary knee problems, specifically secondary knee injuries^6^ and early-onset knee osteoarthritis.^31^ Despite a generally high return-to-play rate for female players (70%),^1,34^ for many (20%-30%) in the WNBA, it is a career-ending injury.^1,3^ For those who do return to play, they experience a shortened career (4.8 vs 8.1 years)^20^ and reduced levels of competitive performance.^3^

An understanding of the epidemiology and cause of an injury is crucial in designing programs to mitigate the injury risk.^23,35^ Although many approaches are available to support an increased understanding of ACL injury mechanisms,^19^ video analysis is a frequently used and valid tool to investigate injury mechanisms, playing situations, and gross biomechanics preceding and during actual injuries.^19^ Several video analysis studies of ACL injuries have been performed across different sports.** One recent article performed a systematic video analysis of ACL injuries in male basketball players; however, the authors did not include female players.^33^ That article reported differences in contact mechanisms and situational patterns compared with earlier research on male players,^17^ with a higher proportion of contact at the time of injury (61% vs 29%) and a lower rate of direct contact injuries (3% vs 24%). Furthermore, injuries were more commonly associated with offensive possession (69%), whereas the previous study highlighted defensive actions as the primary context (74%). The only research on female basketball players includes a study performed 17 years ago^17^ with a small sample size, which may not be valid in current times, given the changes seen for male ACL injuries,^33^ as well as a more recent study with a lack of focus on biomechanics at the time of injury.^1^ There is a need to replicate recent research on male players in female players as a matter of equality and diversity in this field of research. Findings would have strong implications for the design of programs to mitigate the injury risk.

This study aimed to clarify the mechanisms, situational patterns, and kinematics involved in ACL injuries among professional female basketball players. The secondary purpose was to elucidate the distribution of ACL injuries across different phases of the game, court locations, and the player’s position. By specifying this information, we aimed to provide valuable insights that can enhance the design of more effective injury prevention programs.

## Methods

### Injury Identification and Video Extraction

A systematic search was performed across 5 seasons (from 2018/2019 to 2022/2023), using available online resources, to identify ACL injuries in female professional basketball teams. Included in the search were the top leagues of France (Ligue Féminine de Basketball), Germany (1. Damen Basketball Bundesliga), Italy (Lega Basket Femminile Serie A1), Spain (Liga Femenina Endesa), and Sweden (Svenska Basketligan Dam).

All videos and information on the ACL injury were accessible to the public at the time of collection. No personal information was acquired; therefore, no ethical permission was required. All data retrieved were handled with confidentiality.

The study’s methodology has previously been described.^33^ In summary, to identify ACL injuries, each season’s team rosters were extracted from online databases (www.eurobasket.com; www.basketlfb.com; www.toyota-dbbl.de; www.legabasketfemminile.com; www.feb.es; www.sbldam.se) and individual team websites. Each team and player were combined in subsequent searches on www.google.com using the keywords “anterior cruciate ligament,” “korsbandsskada,” “ligamento cruzado anterior,” “ligament croise,” “legamento crociato anteriore,” and “kreuzbanddriss.” Additionally, the same systematic approach was extended to other data sources, including national and local media, to identify potential injuries that might have been overlooked. Injuries were only considered for analysis if information on the nature of the injury was clear. All players currently under contract with teams in the specified leagues were included, allowing injuries that occurred during cup games, national team competitions, and junior team competitions to be included.

Videos of games were obtained from online digital platforms (www.youtube.com; www.sbldamplay.se; www.sporttotal.tv; www.lbftv.it; www.canalfeb.tv) available to the public. When a video was unavailable, additional searches were conducted on other platforms (eg, local media, club social media channels). Videos of the ACL injuries were edited to include approximately 10 to 15 seconds before and 3 to 5 seconds after the estimated injury frame (IF), allowing a thorough evaluation of the circumstances leading up to the injury and the injury mechanism itself.

### Video Evaluation

There were 3 independent reviewers, including 2 experienced sports medicine physicians (F.T. and F.D.V.) and a medical student with experience in professional men’s basketball on a national and international level (K.H.T.). In cases in which findings varied between reviewers, discrepancies were resolved through a consensus. Each video was downloaded onto a personal computer and viewed with open-source Kinovea software (Version 0.9.5), which has been shown to provide reliable angular measurements in a previous study.^27^ Depending on availability, videos contained 1 to 3 camera angles. The videos were evaluated using 2 predetermined checklists (see the Appendix, available in the online version of this article).

Each reviewer evaluated the original video to define the playing phase, characterized as defensive or offensive, based on ball possession and specific playing situation. A series of views were then used to determine the injury mechanism and situational pattern. The injured knee was determined based on injury history gathered and video analysis. Leg loading was established on the injured limb, uninjured limb, or both limbs. Subsequently, the intensity of action was determined based on estimated horizontal and vertical velocities (zero, low, moderate, and high). These categories were defined subjectively, with a consensus reached among the reviewers based on observed patterns of velocity in the videos. Overall, 3 injury mechanism categories were used according to previous research^33,36^: (1) noncontact, defined as an injury occurring without any contact (at the knee or any other level) before or at the IF; (2) indirect contact, defined as an injury resulting from an external force applied to the basketball player but not directly to the injured knee; and (3) direct contact, defined as an injury resulting from an external force directly applied to the injured knee. We used the term “situational pattern” to determine the playing action and context of the injury. This was utilized exclusively in cases involving either indirect contact or noncontact injuries.

An assessment of neurocognitive perturbation was performed. All videos were analyzed to determine whether neurocognitive errors, specifically faulty inhibitory control, were present. Inhibitory control depends on both motor response inhibition and attentional inhibition. Motor response inhibition was defined as the ability to stop or adjust unwanted or incorrect motor actions in response to situational demands.^10^ In this study, an action was considered “unwanted” if it resulted in a visibly abrupt correction, hesitation, or countermovement, suggesting attempted inhibition of the initial motion. There is a time delay between the stimulus and the action of a correct motor response, with previous studies suggesting a delay of approximately 450 to 1200 milliseconds.^12,32^

Opponents may hide their intention with the purpose of creating an advantage (disguise action) or performing deceptive actions, such as fakes. To assess these responses, an assessment was performed on whether a deceiving action occurred before the injury and to identify the IF at which such an action occurred. The time from the deceiving action to initial contact (IC) was calculated. The responses were classified as neurocognitive perturbation if an action from an opponent created an error in the motor response or if a player’s focus on an external point (eg, looking at the ball, on another player, or the basket) led to a selective attention deficit, resulting in a loss of attention in the neuromuscular control or response.

### Biomechanical Analysis (Kinematics)

Injuries arising from noncontact and indirect contact scenarios underwent comprehensive biomechanical (kinematic) analysis, provided that a clear and acceptable-quality frontal and/or sagittal view was available. Analysis was performed with consideration to alignment in the frontal or sagittal plane at IC with the court and at the IF to accurately assess intersegmental relationships and joint angles. Analysis was performed collectively as a group during the consensus meeting.

Custom-made software (GPEM Screen Editor; GPEM) was used to accurately estimate sagittal- and frontal-plane angles. An estimation to the nearest 5° at IC and the IF was made. The remaining estimated frontal- and coronal-plane joint positions were categorized according to appearance. Foot strike was evaluated according to previous methodology^33,36^ at IC with the ground and at the IF. Variables that have been evaluated are listed in the Appendix.

### Season, Position, Game, and Court Distribution

Season, in-game, and court details were collected from each injury case using structured web research and analysis of the video, relating the injured player to the surroundings. Specifically, data were compiled regarding the player position, month of injury, game phase leading to the injury, number of minutes played in the particular game, and precise court location based on a customized version of a basketball court. The player’s position at the IF was determined through video analysis using pre-existing court lines and spatial references to court markings. The basketball court was divided into 6 zones (See Figure 6).

### Statistical Analysis

Excel (Version 16.82; Microsoft) and SPSS Statistics (Version 29.0.1.1; IBM) were used for analysis. Categorical variables are presented as absolute numbers and percentages, while continuous variables are reported as means ± standard deviations. The chi-square test was used to compare groups within relevant categories. A statistical significance level of *P* < .05 was used.

## Results

**Figure 1.**
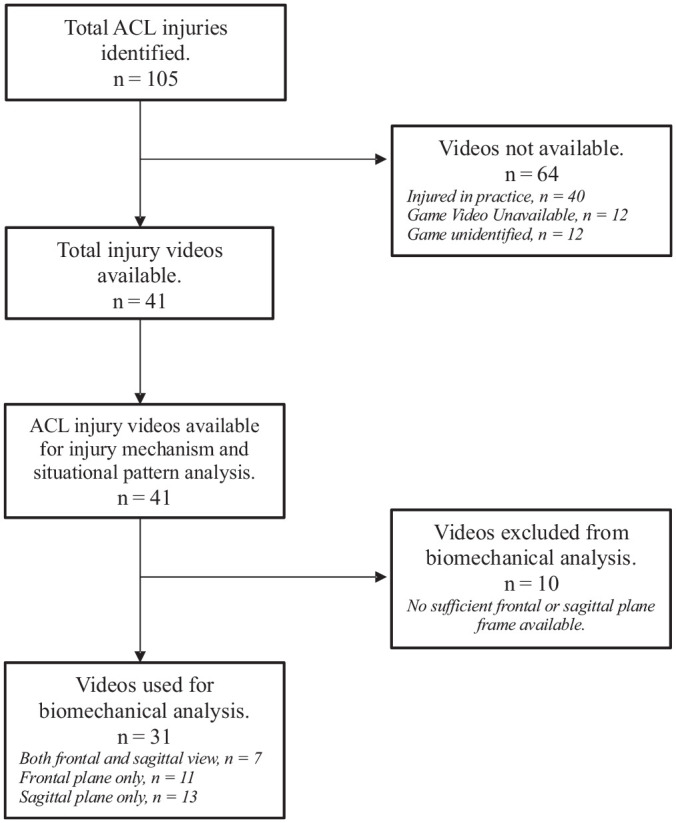
Flowchart of the study.

A total of 105 ACL injuries were identified. Among these, 22 occurred in 1. Damen Basketball Bundesliga (Germany), 15 in Ligue Féminine de Basketball (France), 32 in Lega Basket Femminile Serie A1 (Italy), 24 in Liga Femenina Endesa (Spain), and 12 in Svenska Basketligan Dam (Sweden). Video footage was identifiable and available in 41 cases (39%). The flowchart of the study is presented in Figure 1.

**Figure 2.**
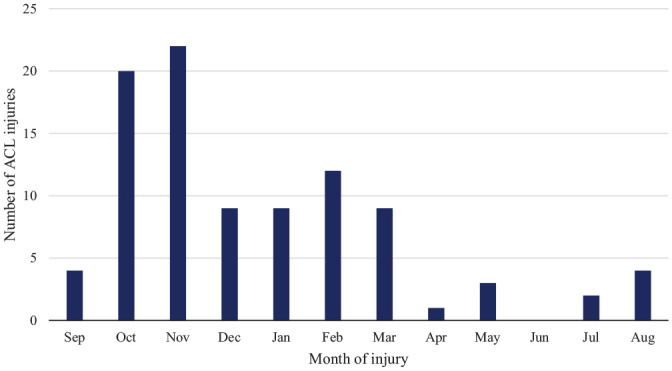
Season distribution of anterior cruciate ligament injuries throughout the basketball season (n = 95). A higher incidence was noted early in the season.

The season distribution (n = 95) showed a higher incidence of injuries early in the season (October-November; n = 42 [44%]), with a comparatively lower frequency during the rest of the season (December-May; n = 43 [45%]). The off-season (June-September) recorded an even lower frequency of injuries (n = 10 [11%]). The season distribution is illustrated in Figure 2.

The mean age of the injured players was 25 ± 4.7 years (n = 41; with video analysis only), with a mean height of 179 ± 6.5 cm (n = 41; with video analysis only). Of the total analyzed injuries, 23 (56%) affected the right ACL, while 18 (44%) affected the left ACL.

### Injury Mechanism

Video footage of sufficient quality and with appropriate angles for injury mechanism and situational pattern analysis was available for 41 cases (39%). More injuries occurred while attacking (n = 28 [68%]) than defending (n = 13 [32%]). Most injuries (n = 33 [80%]) involved loading of the injured leg, with single-leg loading on the ground commonly observed (n = 21 [51%]). We categorized 23 (56%) indirect contact and 18 (44%) noncontact injuries, with no direct contact injuries observed. In situations involving indirect contact, the upper body emerged as the most frequent contact point both at the time preceding the injury (82%) and at the IF (83%). A moderate or high horizontal speed (80%) was more common than a moderate or high vertical speed (20%) at the time of injury (see Table 1 for more details).

**Table 1:** Mechanisms of ACL Injuries (n = 41)^*a*^

	No.
Playing phase before injury	
Offensive	28
Defensive	13
Court location at time of injury	
Zone 1	5
Zone 2	3
Zone 3	3
Zone 4	2
Zone 5	24
Zone 6	4
Player contact preceding injury	
Yes	22
No	16
Unsure	3
If indirect contact, where?	
Upper body	18
Pelvis	3
Unsure	1
Player contact at IF	
Yes	12
No	25
Unsure	4
If indirect contact at IF, where?	
Upper body	10
Pelvis	2
Injury mechanism	
Direct contact	0
Indirect contact	23
Noncontact	18
No. of feet on ground	
1	21
2	12
Missing	8
Leg loading at IF	
Injured leg	33
Uninjured leg	1
Missing	7
Horizontal speed	
Zero	3
Low	3
Moderate	12
High	21
Missing	2
Vertical speed	
Zero	9
Low	22
Moderate	2
High	6
Missing	2
Distance from ball, m	
0-1	23
1-2	7
2-3	6
>3	5

a ACL, anterior cruciate ligament; IF, injury frame.

### Situational Pattern

**Figure 3.**
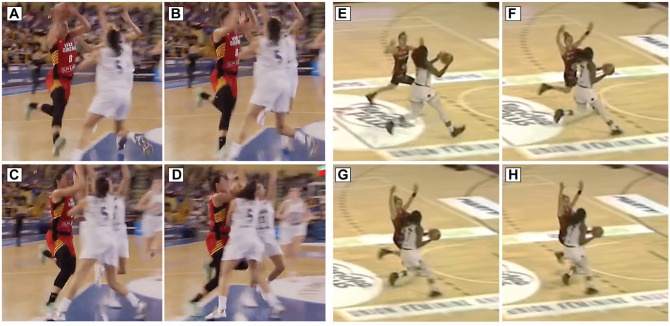
Images of 2 examples of offensive cut anterior cruciate ligament injuries. (A-D) An indirect contact injury. (A) Before contact with the floor. (B) Initial contact with the floor. (C) Estimated injury frame. (D) After injury frame. (E-H) A noncontact injury. (E) Before contact with the floor. (F) Initial contact with the floor. (G) Estimated injury frame. (H) After injury frame.

Overall, 3 main situational patterns, accounting for 95% of injuries, were identified. Offensive cut injuries were the most common, accounting for half (n = 20 [49%]) of all injuries, in which a player with team ball possession executed a change of direction to outmaneuver the defensive opponent (Figure 3). Of these, 10 (50%) occurred with indirect contact, while 9 (45%) were noncontact (1 unidentifiable). Nearly all (90%) involved moderate or high horizontal speeds.

**Figure 4.**
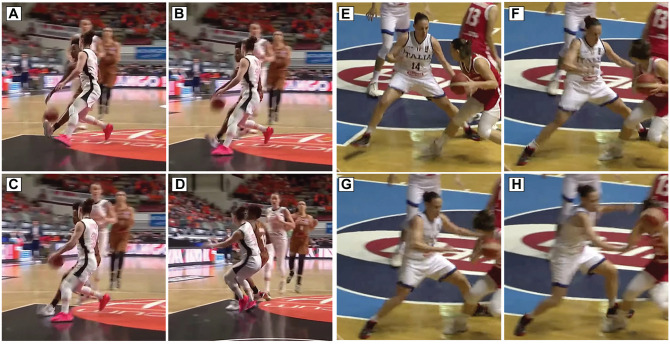
Images of 2 examples of defensive cut anterior cruciate ligament injuries. (A-D) An indirect contact injury. (A) Before contact with the ground (contact with an offensive player in the upper body). (B) Initial contact with the floor. (C) Estimated injury frame. (D) After injury frame. (E-H) A noncontact injury. (E) Before contact with the floor. (F) Initial contact with the floor. (G) Estimated injury frame. (H) After injury frame.

The defensive cut was the second most prominent situational pattern, accounting for almost a third of the injuries (n = 12 [29%]). This was characterized as a defending player using a change of direction to adjust her defensive position in response to her offensive counterpart (Figure 4). Of these, 7 (58%) involved noncontact, while 5 (42%) were indirect contact.

Landing from a jump was the third most common situational pattern, accounting for 1 in 6 injuries (n = 7 [17%]). Most of these were indirect contact (n = 6 [86%]). Sprinting actions accounted for the remaining 2 situations (5%). Additional details can be found in Table 2.

**Table 2:** Situational Patterns of ACL Injuries (n = 41)^*a*^

Pattern	Playing Phase	Injury Mechanism	Horizontal Velocity	Vertical Velocity	Court Location
Offensive cut: 20 (49)	Offensive: 20 (100)	Indirect contact: 10 (50)	Zero: 0 (0)	Zero: 6 (30)	Zone 1: 2 (10)
					Zone 2: 1 (5)
					Zone 3: 1 (5)
			Low: 2 (10)	Low: 14 (70)	Zone 4: 1 (5)
	Defensive: 0 (0)	Noncontact: 9 (45)	Moderate: 5 (25)	Moderate: 0 (0)	Zone 5: 14 (70)
		Missing: 1 (5)	High: 13 (65)	High: 0 (0)	Zone 6: 1 (5)
Defensive cut: 12 (29)	Offensive: 0 (0)	Indirect contact: 5 (42)	Zero: 0 (0)	Zero: 3 (25)	Zone 1: 2 (17)
					Zone 2: 1 (8)
					Zone 3: 2 (17)
			Low: 1 (8)	Low: 7 (58)	Zone 4: 0 (0)
	Defensive: 12 (100)	Noncontact: 7 (58)	Moderate: 5 (42)	Moderate: 2 (17)	Zone 5: 7 (58)
			High: 6 (50)	High: 0 (0)	Zone 6: 0 (0)
Landing from jump (3 unilateral, 3 bilateral, 1 unsure): 7 (17)	Offensive: 6 (86)	Indirect contact: 6 (86)	Zero: 4 (57)	Zero: 0 (0)	Zone 1: 1 (14)
					Zone 2: 1 (14)
					Zone 3: 0 (0)
			Low: 0 (0)	Low: 0 (0)	Zone 4: 1 (14)
	Defensive: 1 (14)	Noncontact: 1 (14)	Moderate: 1 (14)	Moderate: 0 (0)	Zone 5: 2 (29)
			High: 2 (29)	High: 7 (100)	Zone 6: 2 (29)
Other (2 sprinting): 2 (5)	Offensive: 2 (100)	Indirect contact: 1 (50)	Zero: 0 (0)^*b*^	Zero: 0 (0)^*b*^	Zone 1: 0 (0)
					Zone 2: 0 (0)
					Zone 3: 0 (0)
			Low: 0 (0)	Low: 1 (50)	Zone 4: 0 (0)
	Defensive: 0 (0)	Noncontact: 1 (50)	Moderate: 1 (50)	Moderate: 0 (0)	Zone 5: 1 (50)
			High: 0 (0)	High: 0 (0)	Zone 6: 1 (50)

a Data are reported as n (%). ACL, anterior cruciate ligament.

b The second injury is “unsure”.

### Biomechanical Analysis (Kinematics)

Video footage quality and angles were sufficient for biomechanical analysis in 33 cases (80%). There were 8 cases (24%) that included both the frontal and sagittal planes, 13 cases (39%) were limited to the frontal plane only, and 12 cases (36%) were restricted to the sagittal plane. All angle data are reported as median values.

At IC in the sagittal plane, players displayed an upright trunk (5°), a flexed hip (45°), and a slightly flexed knee (35°). The ankle was plantarflexed (–8.5° of dorsiflexion), with a predominantly flat-foot strike appearance (49%). In the frontal plane at IC, the trunk was tilted ipsilaterally (12.5°), typically in a neutral position (36%) with an abducted hip (55%), while knee alignment was predominantly neutral (42%). Foot positioning was primarily externally rotated (39%).

At the estimated IF in the sagittal plane, the trunk remained upright (10°; +5° from IC) with slightly increased hip flexion (55°; +10° from IC), greater knee flexion (55°; +20° from IC), and a neutral ankle position (0°; +8.5° of dorsiflexion from IC) with a planted flat foot (91%). In the frontal plane, the trunk remained tilted ipsilaterally (10°; –2.5° from IC) in neutral (30%) or toward the uninjured side (24%). The hip remained abducted in most cases (46%), with a greater prevalence of knee valgus (64%) and a predominantly externally rotated foot (36%).

A significant increase in hip internal rotation and/or adduction from IC to the IF was seen in half of cases (52%), with valgus collapse occurring in more than 1 in 4 cases (27%). Additional details are reported in Tables 3 and 4.

**Table 3:** Sagittal-Plane Biomechanical Variables of ACL Injuries (n = 33)^*a*^

	Total	Offensive Cut	Landing From Jump	Defensive Cut	Other
Flexion angle, deg					
Trunk at IC	5 (0.25 to 23)	5 (0 to 15)	00 (–5 to 38)	10 (–5 to 20)	20
Trunk at IF	10 (3.75 to 25)	5 (12.5 to 23.75)	–1.5 (2.5 to 2)	15 (1 to 24)	30
Hip at IC	45 (35 to 60)	40 (31.25 to 63.75)	26.5 (19 to 55)	60 (35 to 60)	85
Hip at IF	55 (33.75 to 70)	40 (30 to 82)	55 (21.5 to 65)	60 (36.25 to 68.75)	80
Knee at IC	35 (15 to 40)	30 (11.25 to 40)	35 (17.5 to 37.5)	40 (15 to 50)	95
Knee at IF	55 (35 to 66.25)	45 (32.75 to 73.75)	55 (35 to 58.5)	60 (37.75 to 68.75)	90
Ankle at IC	–8.5 (–11.5 to 13.75)	–7.5 (–17.25 to 17.5)	–5 (–10 to 0)	–10 (–10 to 15)	15
Ankle at IF	00 (–1 to 20)	00 (–12.5 to 25)	10 (–2.75 to 15)	5 (0 to 20)	15
Foot strike at IC					
Heel	12 (36)	8 (57)	00 (0)	4 (33)	00 (0)
Flat	16 (49)	5 (36)	4 (67)	6 (50)	1 (100)
Toe	5 (15)	1 (7)	2 (33)	2 (17)	00 (0)
Unsure	00 (0)	00 (0)	00 (0)	00 (0)	00 (0)
Foot strike at IF					
Heel	00 (0)	00 (0)	00 (0)	00 (0)	00 (0)
Flat	30 (91)	12 (86)	6 (100)	11 (92)	1 (100)
Toe	3 (9)	2 (14)	00 (0)	1 (8)	00 (0)
Unsure	00 (0)	00 (0)	00 (0)	00 (0)	00 (0)

a Data are reported as median (interquartile range) or n (%). ACL, anterior cruciate ligament; IC, initial contact; IF, injury frame.

**Table 4:** Frontal- and Transverse-Plane Biomechanical Variables of ACL Injuries (n = 33)^*a*^

	Total	Offensive Cut	Landing From Jump	Defensive Cut	Other
Flexion angle, deg					
Trunk tilt at IC	12.5 (0 to 16.25)	10 (0 to 15)	15 (10 to 20)	7.5 (–5 to 20)	—
Trunk tilt at IF	10 (5 to 20)	10 (1.25 to 13.75)	15 (10 to 20)	16 (1.25 to 28.75)	—
Trunk rotation at IC					
Toward injured limb	6 (18)	1 (7)	1 (17)	4 (33)	00 (0)
Neutral	12 (36)	5 (36)	2 (33)	5 (42)	00 (0)
Toward uninjured limb	4 (12)	2 (14)	2 (33)	00 (0)	00 (0)
Unsure	11 (33)	6 (43)	1 (17)	3 (25)	1 (100)
Trunk rotation at IF					
Toward injured limb	4 (12)	1 (7)	00 (0)	3 (25)	00 (0)
Neutral	10 (30)	3 (21)	3 (50)	4 (33)	00 (0)
Toward uninjured limb	8 (24)	4 (29)	2 (33)	2 (17)	00 (0)
Unsure	11 (33)	6 (43)	1 (17)	3 (25)	1 (100)
Knee alignment at IC					
Valgus	10 (30)	4 (29)	2 (33)	4 (33)	00 (0)
Neutral	14 (42)	7 (50)	1 (17)	5 (42)	1 (100)
Varus	1 (3)	00 (0)	00 (0)	1 (8)	00 (0)
Unsure	8 (24)	3 (21)	3 (50)	2 (17)	00 (0)
Knee alignment at IF					
Valgus	21 (64)	10 (71)	4 (67)	7 (58)	00 (0)
Neutral	5 (15)	1 (7)	00 (0)	3 (25)	1 (100)
Varus	00 (0)	00 (0)	00 (0)	00 (0)	00 (0)
Unsure	7 (21)	3 (21)	2 (33)	2 (17)	00 (0)
Hip alignment at IC					
Abduction	18 (55)	6 (43)	4 (67)	8 (67)	00 (0)
Neutral	6 (18)	00 (0)	1 (17)	4 (33)	1 (100)
Adduction	00 (0)	00 (0)	00 (0)	00 (0)	00 (0)
Unsure	9 (27)	8 (57)	1 (17)	00 (0)	00 (0)
Hip alignment at IF					
Abduction	15 (46)	5 (36)	3 (50)	7 (58)	00 (0)
Neutral	1 (3)	00 (0)	00 (0)	1 (8)	00 (0)
Adduction	2 (6)	2 (14)	00 (0)	00 (0)	00 (0)
Unsure	15 (46)	7 (50)	3 (50)	4 (33)	1 (100)
Foot position at IC					
External	13 (39)	6 (43)	2 (33)	5 (42)	00 (0)
Neutral	6 (18)	2 (14)	1 (17)	3 (25)	00 (0)
Internal	1 (3)	1 (7)	00 (0)	00 (0)	00 (0)
Unsure	13 (39)	5 (36)	3 (50)	4 (33)	1 (100)
Foot position at IF					
External	12 (36)	4 (29)	3 (50)	5 (42)	00 (0)
Neutral	4 (12)	2 (14)	00 (0)	2 (17)	00 (0)
Internal	4 (12)	2 (14)	1 (17)	1 (8)	00 (0)
Unsure	13 (39)	6 (43)	2 (33)	4 (33)	1 (100)
Hip internal rotation/adduction from IC to IF					
Yes	17 (52)	7 (50)	5 (83)	5 (42)	00 (0)
No	9 (27)	3 (21)	00 (0)	5 (42)	1 (100)
Unsure	7 (21)	4 (29)	1 (17)	2 (17)	00 (0)
Valgus collapse					
Yes	9 (27)	2 (14)	5 (83)	1 (8)	1 (100)
No	18 (55)	7 (50)	1 (17)	10 (83)	00 (0)
Unsure	6 (18)	5 (36)	00 (0)	1 (8)	00 (0)
Neurocognitive perturbation (n = 41)					
Yes	23 (56)	7 (35)	5 (71)	11 (92)	00 (0)
No	18 (44)	13 (65)	2 (29)	1 (8)	2 (100)

a Data are reported as median (interquartile range) or n (%). ACL, anterior cruciate ligament; IC, initial contact; IF, injury frame.

### Neurocognitive Analysis

Video footage quality and angles were sufficient for neurocognitive analysis in 41 cases (100%). Of these videos, 23 (56%) displayed signs of neurocognitive perturbation, and 18 (44%) had no such signs. The mean time from perturbation to IC was 510 milliseconds. In defensive situations, 12 players (92%) experienced neurocognitive errors before the injury, and in offensive situations, 11 players (39%) experienced such errors. Of the noncontact injuries evaluated, 13 players (72%) displayed errors, while among the indirect contact injuries, 10 players (44%) displayed signs of errors. Further details are presented in Table 5.

**Table 5:** Neurocognitive Perturbation (n = 41)^*a*^

	Yes	No	*P* Value
Total	23 (56)	18 (44)	.435
Offensive phase (n = 28)	11 (39)	17 (61)	.257
Defensive phase (n = 13)	12 (92)	1 (8)	.**002**
Indirect contact (n = 23)	10 (44)	13 (57)	.532
Noncontact (n = 18)	13 (72)	5 (28)	.059

a Data are reported as n (%). *P* values were calculated with the chi-square test. Bold value indicate statistical significance.

### Position, Game, and Court Distribution

Data according to player position (n = 41), match timing (n = 38), and court location (n = 41) were available. A total of 19 injuries (46%; *P* < .001) occurred in small forwards, 11 (27%) in shooting guards, 6 (15%) in point guards, 4 (10%) in power forwards, and 1 (2%) in centers. The backcourt positions (point guard, shooting guard, and small forward) accounted for the vast majority of the injuries (n = 36 [88%]).

**Figure 5.**
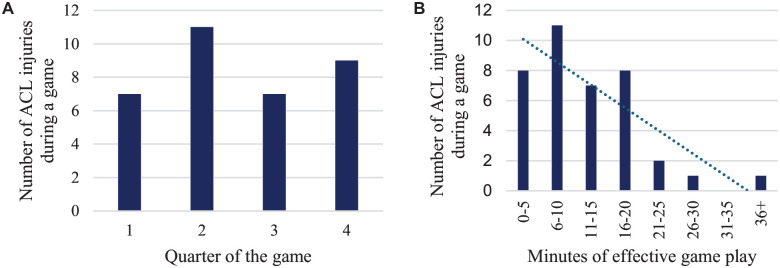
(A) Distribution of injuries per quarter of a basketball game (n = 34). (B) Distribution of effective minutes played by the athlete during the game when the injury occurred (n = 38).

There were 18 ACL injuries (53%) that occurred in the first half of the game and 16 (47%) in the second half. We observed 7 injuries (21%) in the first quarter, 11 (32%) in the second quarter, 7 (21%) in the third quarter, and 9 (26%) in the fourth quarter (Figure 5A). Analysis of players’ actual playing time when correcting for substitutions was possible in 38 cases. The majority (n = 34 [89%]) of the injuries occurred within the initial 20 minutes of effective game play, with half of the injuries (n = 19 [50%]) taking place in the first 10 minutes and one-fifth of the injuries within the first 5 minutes (n = 8 [21%]) (Figure 5B). On average, ACL injuries occurred after 12.3 ± 7.7 minutes of effective playing time.

**Figure 6.**
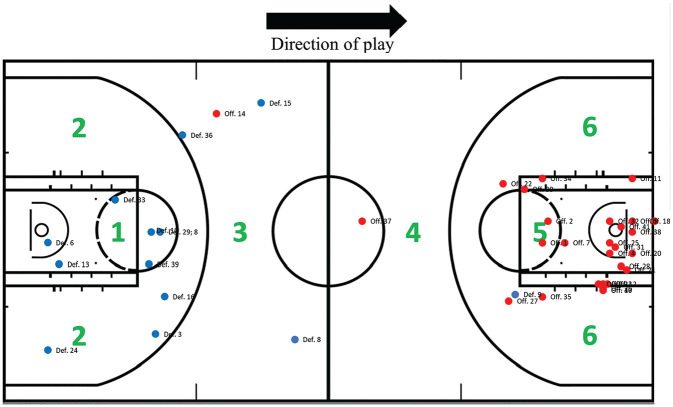
Distribution of the location of the court where anterior cruciate ligament injuries occurred. The red dots indicate team offensive possession, and the blue dots indicate team defensive possession.

Figure 6 illustrates the court location of injuries. The most common location in which the injury occurred was zone 5 (n = 24 [59%]), with the second most common location being zone 1 (n = 5 [12%]). A comprehensive list is provided in Table 1.

## Discussion

The key findings of this study are that (1) all identified ACL injuries in female basketball were either indirect contact or noncontact, with indirect contact being the predominant mechanism; (2) more than two-thirds of injuries occurred in offensive situations; (3) 3 main situational patterns were identified, accounting for 95% of all injuries: offensive cut, defensive cut, and landing from a jump; and (4) notable differences were observed across player positions, game quarters, court locations, and phases of the season.

### Injury Mechanism

More than two-thirds (68%) of ACL injuries in female basketball occurred during offensive possession, consistent with previous basketball research (74%-85%).^5,17^ Interestingly, this differs distinctly from other sports, such as soccer (30%), in which injuries predominantly occur during defensive possession.^18,36^

All injuries occurred without direct contact with the injured knee, with the main mechanism being indirect contact (56%). This aligns with previous studies emphasizing the predominant occurrence of indirect contact or noncontact injuries.^††^ Previous research has depicted a rather diverse pattern of injury mechanisms among basketball players with ACL injuries, particularly in men. One study suggested that noncontact injuries accounted for a small part (19%) in men’s basketball while indirect contact injuries formed the majority (78%).^25^ Another study suggested a similar pattern to the injuries in our study (39% noncontact vs 58% indirect contact).^33^ This is different compared to studies on female basketball players in whom noncontact injuries accounted for the bulk of the injuries (65%) while indirect contact injuries were less common (23%).^30^

### Situational Pattern

Most ACL injuries occurred during 3 main situational patterns: (1) offensive cut (49%), (2) defensive cut (29%), and (3) landing from a jump (17%). In contrast to an earlier study on female players, we found a higher incidence of injuries during cutting (78% vs 9%) and less when landing from a jump (17% vs 59%).^17^ We reported a similar proportion of offensive cutting injuries (49%) to our recent work on male basketball players (47%),^33^ reinforcing this as the dominant situational pattern for ACL injuries in elite basketball. Similar to previous research,^33^ we found that around half of these offensive cutting injuries involved contact before the IF, mostly at the upper body (82% of indirect contact cases). The low incidence of landing injuries (17%) is in contrast to previous research in basketball (78%), which reported landing as the main situation in which injuries occurred.^17^

In a study by Krosshaug et al,^17^ the main pattern for female basketball players was landing from a jump (59%), proposing that most landing injuries occur as noncontact injuries, primarily with both feet planted. One recent study on professional male basketball players observed that landing from a jump was the second most common pattern for ACL injuries and that most of the injuries (88%) occurred with a single foot on the floor when landing from a jump.^33^ We noted an even split between players having 1 or 2 feet planted on the ground at the IF. In our study, the majority (86%) of these injuries occurred during offense, with an equal number (86%) resulting from indirect contact. In all cases of indirect contact, the upper body experienced contact. Why this difference between men and women is seen is unknown. The landing injuries underscore the importance of risk reduction programs focusing on neuromuscular control, mostly during rebound attempts and layups with contact, as players typically focus on the task during a game rather than have an awareness of surrounding players. Why a difference between mainly landing injuries to mainly cutting injuries is observed is unclear, but we suggest that it is because of a higher tempo in basketball today, with more frequent cuts.

Most earlier studies on basketball players did not distinguish between offensive and defensive cutting injuries.^1,17,25,30^ We found that defensive cut was the second most prominent situational pattern, with a higher prevalence (29%) than in our study on male players (14%).^33^ The defensive cut is well recognized as a high-risk pattern in other sports, such as soccer and rugby, often occurring in pressing situations.^8,18,36,37^ While defensive cut injuries were more frequently noncontact (58%), an indirect contact injury often involved contact primarily to the upper body and/or the pelvis (100%).

### Biomechanical Analysis

Our kinematic data align with the established understanding of ACL injuries in basketball players, consistently revealing a knee-dominant pattern at the time of injury.^1,17,33^ Early knee flexion at IC (35°) was indicative of potentially high ACL loading and a vulnerable position, contributing to the risk of injuries. It is also noteworthy that this angle is higher than those reported in earlier studies (13°-24°).^5,13,16,17,33^ A recent study by Zago et al^39^ analyzed the biomechanics of noncontact injuries in male soccer players. The flexion angles were higher than previously found (42°), suggesting that higher knee angles are present in cutting movements and landing tasks in an attempt to reduce body momentum.

While the trend of increasing knee flexion from IC to the IF has been noted previously, the angle at the IF in our study (55°) is higher than previously found in basketball studies (27°-47°).^13,16,17,33^ However, this aligns with recent video analyses in other sports (35°-53°).^8,18,36,39^ When comparing the results of our study with those from a previous study investigating female soccer players,^18^ there are similar values of knee flexion during injuries both at IC (30° in soccer vs 35° in this study) and the IF (52.5° in soccer vs 55° in this study).

The ankle dorsiflexion change from IC to the IF (–8.5° to 0°; +8.5°) is consistent with research on ACL injuries in other sports (0°-13°).^8,18,36^ Boden et al^5^ described a similar pattern, noting a relatively small change in ankle angles (4°) from IC to the IF compared with healthy controls (44° change). Their study suggested that inadequate ankle flexion kinematics could lead to inefficient absorption of ground-reaction forces, making the knee a more significant force reduction site, thereby creating a vulnerability for motion such as internal rotation rather than flexion.^5^ At the IF, the majority of their injuries (80%) involved a flat-foot strike. This, coupled with the minimal change in ankle dorsiflexion, likely contributes to the high forces placed on the ACL by impeding the calf muscles from effectively absorbing the forces.^5^

Although the hip was flexed at IC (45°) and remained flexed (55°) at the IF, hip flexion at both IC (35°-42°) and the IF (37°-55°) is recognized as a high-risk movement in basketball and other sports. Female athletes, in general, tend to exhibit higher hip flexion angles when injured, as supported by the existing literature.^5,8,13,17,18,36^

The data indicate that knee valgus was prevalent (64% at IF) and poses a high risk for female basketball players, consistent with previous research findings.^1,8,25,36^ We also observed that hip abduction was common, and clinically significant hip internal rotation/adduction from IC to the IF was noted, also aligned with earlier research.^1,17,25^

### Neurocognitive Analysis

Basketball research has not extensively explored neurocognitive disturbances and their associated risks. One recent article examined the role of neurocognitive perturbation in noncontact ACL injuries in soccer.^14^ Our data reveal that 56% of all ACL injuries in female basketball involved neurocognitive perturbation in the time leading up to the injury, with 72% of noncontact injuries exhibiting such perturbation. Intriguingly, 92% of the defensive injuries involved neurocognitive perturbation. Furthermore, in the limited field of basketball, our data reveal that ACL injuries occurred in close proximity (0-1 m) to both the ball (68%) and an opponent (65%), a trend supported by existing literature.^5,17^ When experiencing neurocognitive perturbation, the mean time from the deceiving action to IC was 510 milliseconds, almost double the time reported in the previously mentioned article (256 milliseconds).^14^ Further research in this area is recommended to investigate the mechanisms and patterns.

### Season, Position, Game, and Court Distribution

A substantial proportion of injuries (44%) occurred in the first 2 months of the season, deviating from other studies suggesting a bimodal pattern.^18,36^ The early pattern indicates that this may be attributed to insufficient preparedness during the transition from the preseason to early season. We suggest that intense preseason training poses a risk of accumulating fatigue, which could increase the risk of ACL injuries in the early season.

When breaking down the data by position, a clear pattern emerges, with backcourt players being more prone to ACL injuries. We suggest that the backcourt positions generally have a more driving-prone playing style as well as a playing style with higher horizontal velocity. This aligns with previous findings indicating that guards are predominantly injured (53%).^1^ A study on National Basketball Association (NBA) players highlighted that players with a greater tendency to drive the ball face a higher ACL injury risk compared with those with a lower tendency (5.7% vs 3.8%).^28^ This is attributed to a playing style that leans more toward high-risk movements, such as quick lateral movements and acceleration/deceleration.^28^

A notable insight from our court distribution analysis is that 59% of the ACL injuries occurred in the offensive 3-second area (area 5), an area of the court characterized by high player density, dynamic movements, and frequent contact. This is somewhat different from male basketball players, who have even more ACL injuries within area 5 (73%).^33^

Our study shows a similar percentage for the first (53%) and second (47%) halves of the game, differing somewhat from earlier studies (62% in second half^15^) but identical with rates in male basketball.^33^ While previous research has suggested fatigue as a risk factor,^15^ our study indicates that this may not be true in professional women’s basketball mainly because of the mean accumulated playing time when injured (12.3 minutes). When looking at the minutes played when injured, a clear trend emerges: players were more likely to be injured within the first 10 minutes played (50%) and the first 20 minutes (89%). This aligns with earlier studies in the NBA (16.5+-11.8)^15^ and studies on male basketball players in European leagues (52% within 10 minutes).^33^ The reason for this pattern is not entirely clear. Still, one suggestion is that substitutes during the game are less likely to be sufficiently warmed up and may not be neurocognitive ready. Furthermore, we do not consider the intensity of the game as a risk factor. Despite the data, the possibility of accumulated fatigue as a risk factor cannot be entirely ruled out, and the potential for acute fatigue should not be overlooked. Moreover, a relatively similar incidence in the quarters of a game was observed (21% in first, 32% in second, 21% in third, and 26% in fourth quarter). Previous research has observed a higher incidence in the fourth quarter (40%).^15^ Our data suggest that the specific quarter does not correlate with an increased injury risk.

The long-standing debate in basketball about the high risk of advertising signs has not been studied until now. Our data show that 13% of the ACL injuries occurred on advertising signs, and more notably, 51% occurred on a painted area (8% of these cases were combined advertising signs and painted floors). However, the painted areas are closely associated with the high-risk areas of the court (areas 1 and 5), possibly linked to high horizontal velocity when entering, the presence of opponents, and neurocognitive perturbation. Further research might bring more light on the claims; however, we firmly believe that there is no relevance in this claim.

### Practical Implications

The findings of this study indicate that many ACL injuries among professional female basketball players may be preventable, as no direct contact injuries were identified. The high prevalence of noncontact injuries (44%) underscores the critical need for targeted and effective injury prevention programs.^2,35^ Developing a successful injury prevention program requires a thorough understanding of the mechanisms behind ACL injuries. We suggest emphasizing both kinematic factors and neurocognitive function during cutting actions, both offensively and defensively. Additionally, improving horizontal deceleration techniques should be a priority for female basketball players.

Injury prevention programs should emphasize proper techniques during cutting actions, particularly those involving rapid deceleration and changes of direction. Incorporating neurocognitive training into common basketball drills may enhance proprioception, decision-making under pressure, and reaction time during dynamic movements in both offensive and defensive settings. Deceleration is a crucial component of preventive training, focusing on horizontal deceleration techniques and landing mechanisms.

Previous research has demonstrated that teaching proper techniques for cutting movements can effectively reduce external knee abduction moments.^9^ Furthermore, changes in kinematics observed during screening for direction changes have been prospectively linked to a higher risk of ACL injuries in a small cohort of female soccer players.^11^

Notably, 89% of ACL injuries in female basketball players occurred within the first 20 minutes of effective playing time. Given the importance of neurocognition and physical readiness, we recommend incorporating a brief in-game warm-up routine to further enhance players’ preparedness.

### Strengths

Our study has several key strengths: (1) It features the largest systematic video analysis sample of ACL injuries in professional female basketball players, surpassing a similar study that examined 39 players (17 male and 22 female).^17^ (2) This study analyzed consecutive injuries. (3) We performed biomechanical (kinematic) analysis using precise measurement tools and 3 independent reviewers. (4) This study included data on the distribution of injuries across various court locations and phases of the game.

### Limitations

The study has some limitations, primarily related to the methodology used to identify ACL injuries. Unlike the gold standard in prospective studies, which involves regular contact with teams, our approach limits the collection of detailed information on concomitant injuries. Instead of using a model-based image-matching technique, which is considered the gold standard, we relied on video analysis to determine kinematics before and at the time of injury.^19^ Despite this, video analysis is a validated method^20^ and is frequently used in similar studies.^7,9,11,36^ Our sample is exclusively composed of European professional female players, which may affect the generalizability of our findings to male athletes and to those at lower levels of competition. Further research is necessary to better understand the injury mechanisms within these other populations.

## Conclusion

In women’s professional basketball, indirect contact as opposed to noncontact was the main ACL injury mechanism. There were 3 main situational patterns observed, with offensive cut being the most common. More ACL injuries occurred in the first 10 minutes of a player’s effective playing time, inside the 3-second area of the court and among small forwards.

## Supplemental Material

sj-pdf-1-ajs-10.1177_03635465251330007 – Supplemental material for A Systematic Video Analysis of Anterior Cruciate Ligament Injuries in Professional Female Basketball Players Supplemental material, sj-pdf-1-ajs-10.1177_03635465251330007 for A Systematic Video Analysis of Anterior Cruciate Ligament Injuries in Professional Female Basketball Players by Kristian Heder Ternell, Filippo Tosarelli, Matthew Buckthorpe, Kristian Samuelsson, Eric Hamrin Senorski and Francesco Della Villa in The American Journal of Sports Medicine

## Footnotes

** References 1, 4, 5, 7, 8, 14, 17, 18, 21, 25, 29, 36, 37, 38.

†† References 1, 4, 7, 8, 17, 18, 21, 24, 25, 29, 36.
