# Supplementary material for: A Systematic Video Analysis of Anterior Cruciate Ligament Injuries in Professional Female Basketball Players
Source: Am J Sports Med. 2025 Apr 6;53(6):1368–80. doi: 10.1177/03635465251330007 (PMC12044211; doi:10.1177/03635465251330007)
Supplement: sj-pdf-1-ajs-10.1177_03635465251330007 – Supplemental material for A Systematic Video Analysis of Anterior Cruciate Ligament Injuries in Professional Female Basketball Players [file sj-pdf-1-ajs-10.1177_03635465251330007.pdf]

Checklist for Biomechanical evaluation

**Variables**	**Categories**
Trunk Flexion at IC and IF<br>(+ flexion, - extension)	Estimation nearest to 5°
Hip Flexion at IC and IF<br>(+ flexion, - extension)	Estimation nearest to 5°
Knee Flexion at IC and IF<br>(+ flexion, - extension)	Estimation nearest to 5°
Ankle Flexion at IC and IF<br>(+ dorsiflexion, - plantarflexion)	Estimation nearest to 5°
Foot Strike at IC and IF	Heel/Flat/Toe/Unsure
Trunk Tilt at IC and IF<br>(+ ipsilateral, - contralateral)	Estimation nearest to 5°
Trunk rotation at IC and IF	Towards injured leg/Neutral/Towards uninjured leg/Unsure
Hip Alignment at IC and IF	Abducted/Neutral/Adducted/Unsure
Knee Alignment at IC and IF	Valgus/Neutral/Varus/Unsure
Foot Position at IC and IF	Externally rotated/Neutral/Internally rotated/Unsure
Significant Hip IR/ADD from IC to IF?	Yes/No/Unsure
Valgus Collapse	Yes/No/Unsure

Checklist for Video evaluation

**Variables**	**Categories**
Injured side	Right/Left
Playing phase before injury	Defensive/Offensive
Court location at injury	1-6
Player situational pattern	Change of direction/Landing from jump/Finish around the rim/Stopping/Initiating jump/Pivoting/Rebounding/Regain balance after layup/Receiving the ball
Player contact preceding injury	Yes/No
- If contact, where?	Upper Body/Pelvis/Injured Leg/Un-injured Leg
Player contact at IF	Direct Contact/Indirect Contact/Non-Contact
- If indirect contact at IF, where?	Upper Body/Pelvis/Injured Leg/Un-injured Leg
Injury Classification	Direct contact/Indirect contact/non-contact
How many feet on the ground	One/Two/Unsure
Leg loading at IF	Injured Leg/Un-injured Leg/Unsure
Horizontal Speed	Zero/Low/Medium/High/Very High/Unsure
Vertical Speed	Zero/Low/Medium/High/Very High/Unsure
Injury occurred on an ad-sign or painted part of court?	Yes, Ad-sign/Yes, painted floor/Yes, both ad-sign and painted floor/No
Did the injury occur after a neurocognitive perturbation?	Yes/No/Unsure
- If yes, at what time? Difference to IC?	Time.
Distance from ball	0-1/1-2/2-3/ >3
Distance from opponent	0-1/1-2/2-3/ >3
